# IRB challenges in multisite studies: A case report and commentary from the Intellectual Disability Supplement to the Irish Longitudinal Study on Ageing (IDS-TILDA)

**DOI:** 10.12688/hrbopenres.13854.1

**Published:** 2024-02-12

**Authors:** Darren McCausland, Margaret Haigh, Philip McCallion, Mary McCarron

**Affiliations:** 1 Trinity Centre for Ageing and Intellectual Disability, The University of Dublin Trinity College, Dublin, Leinster, Ireland; 2 Temple University, Philadelphia, Pennsylvania, USA

**Keywords:** research ethics, health research, multi-site study, institutional review board (IRB)

## Abstract

A shift from single to multi-site health studies enabled a range of research benefits including faster recruitment of larger and more diverse samples; increased statistical power, greater rigour, generalisability, and external reliability; and increased likelihood of impacting policy and clinical practice. However, ethical review of multi-site studies by Institutional Review Boards (IRBs) raises specific challenges compared with single site studies, with requirements to apply to multiple local IRBs increasing the burden on research, possibly endangering the integrity of the research process or inhibiting development of multi-site studies. The option of a single centralised IRB may offer a clearer, more consistent and efficient review process. This study presents a case report and commentary from 15 years engaging with IRBs in multiple sites in Ireland by the Intellectual Disability Supplement to the Irish Longitudinal Study on Ageing (IDS-TILDA). It examines the ethics review process for IDS-TILDA through its first four waves.

While the majority of 48 IRBs granted ethical approval within 13 weeks, six IRBs took 21–47 weeks to approve, leading to delays in data collection of up to 11 months. Despite additional review time, no changes were required to the study protocol. Therefore, a critical impact of the process was the delay in starting data collection within a small number of organisations, and reduced involvement in the study for one organisation. The ethical review process with multiple IRBs increased the degree of complexity of the process, with added bureaucracy and far greater communication required across 48 IRBs, substantially adding to the resource commitment for the review process. The relatively quick approval from the majority of IRBs was partially a result of the longitudinal study building relationships with organisations throughout multiple waves. That other health studies may not accrue this benefit supports calls for a single IRB system for multi-site health studies.

## Introduction

The end of the twentieth century saw a change in health research, with a shift away from studies performed in single institutions to larger multi-site studies involving multiple partners in diverse geographical locations ( Gold & Dewa, 2005). A number of key benefits of multi-site study were behind this shift, including the opportunity they present to recruit larger and more diverse study samples ( Beischel *et al.*, 2016; Flynn, 2009; Smith *et al.*, 2019), and to recruit these samples quicker than is possible in single site studies ( Beischel *et al.*, 2016; Flynn, 2009; Smith *et al.*, 2019; Weinberger *et al.*, 2001). As a consequence, multi-site studies also benefit from the potential for increased statistical power, greater rigor, generalisability, and external reliability ( Beischel *et al.*, 2016; Cohen, 1977; Flynn, 2009; Smith *et al.*, 2019; Weinberger *et al.*, 2001). These improved study properties also enhance the potential impact of multi-site studies and increase the likelihood of their findings influencing policy and clinical practice changes ( Smith *et al.*, 2019).

Ethical review of all research involving human beings is essential to ensure that the welfare, dignity, and rights of research participants are protected ( World Health Organisation, 2023). Many existing ethical codes for research stem from the Nuremburg Code, drafted in 1947 for the Nuremburg Trials ( U.S. Government Printing Office, 1949), and the Helsinki Declaration first adopted in 1964 by the World Medical Association ( World Medical Association, 1964). The framework for current ethical regulations for research in the United States was provided by the Belmont Report published in 1979 ( American Psychological Association, 2008). The report outlined the basic ethical principles of respect for persons, beneficence, and justice, and established applications of these basic principles including informed consent, assessment of risks and benefits, and the selection of subjects ( The National Commission for the Protection of Human Subjects of Biomedical and Behavioral Research, 1979). In Ireland, prior to the recent General Data Protection Regulation (GDPR) ( European Union, 2016), the basis for fundamental rights of research participants may be traced to the Irish Constitution’s expression of equality and protection of persons including their good name ( Government of Ireland, 1937) and to subsequent legislation such as the Data Protection Acts 1988 and 2003 ( Government of Ireland, 1988; Government of Ireland, 2003).

An Institutional Review Board (IRB) is “the institutional entity charged with providing ethical and regulatory oversight of research involving human subjects, typically at the site of the research study” ( National Institutes of Health, 2023). This is typically called a Research Ethics Committees (REC) in Ireland. While essential, ethical review of multi-site studies by IRBs may present specific challenges compared with review for single site studies. In particular, it has been reported that ethical review using multiple local IRBs may not be conducive to multi-site studies, and may be a burden on research or even endanger the integrity of the research process ( Gold & Dewa, 2005; Menikoff, 2010; Silberman & Kahn, 2011; Smith *et al.*, 2019).

Against this, the option for multi-site studies to use a single, centralised IRB has been identified as offering a clearer and more consistent ethical review process ( Driscoll *et al.*, 2008) and facilitating a more streamlined and efficient ethical review process for multi-site research ( Marquis-Gravel *et al.*, 2021; Nichols *et al.*, 2019). However, despite the existence of policy and other legal guidance supporting the use of single IRB systems, recommended approaches have proven difficult to implement in different jurisdictions including the US ( Marquis-Gravel *et al.*, 2021; Nichols *et al.*, 2019) and Australia ( Driscoll *et al.*, 2008; Samir *et al.*, 2022). As a consequence, there remains significant challenges to conducting multi-site studies even where systems are in place for a centralised review process ( Brown *et al.*, 2008; Samir *et al.*, 2022).

### Specific challenges and impacts of ethics processes for multi-site studies

Applications for approval to multiple IRBs increase the complexity of the ethical review process and add to the bureaucracy associated with obtaining approval ( Driscoll *et al.*, 2008; Marquis-Gravel *et al.*, 2021; Samir *et al.*, 2022; Smith *et al.*, 2019). Increased complexity may further impact the process by making communication more challenging for researchers ( Smith *et al.*, 2019). Rather than being supportive, the increased complexity of the ethics process may actually be a barrier to conducting ethical research ( Samir *et al.*, 2022).

Another challenge associated with application processes involving multiple IRBs is the time taken to obtain full approval. While multi-site studies may speed up the recruitment process, one of the critical challenges of multiple IRB ethical approval is the impact that the prolonged process may have on project timeframes, with particular implications for initiating the research process ( Driscoll *et al.*, 2008; Gold & Dewa, 2005; Greene & Geiger, 2006; Marquis-Gravel *et al.*, 2021; Racine & Bracken-Roche, 2019; Salt, 2019; Samir *et al.*, 2022; Smith *et al.*, 2019). The impact for one national study was estimated at a six-eight month delay in the research ( Driscoll *et al.*, 2008), while others were concerned that delays in the process led to a loss of faith in research amongst communities ( Brown *et al.*, 2008). Another practical challenge associated with multiple IRB applications is the direct impact they have on project costs and resource implications related to applying to multiple different individual institutions ( Gold & Dewa, 2005; Greene & Geiger, 2006; Salt, 2019; Smith *et al.*, 2019).

Another critical challenge highlighted in the literature, with a number of different possible implications, relates to the variability and inconsistency involved in ethical review processes across different IRBs ( Driscoll *et al.*, 2008; Gold & Dewa, 2005; Marquis-Gravel *et al.*, 2021; Salt, 2019). This may stem in the first place from inconsistent ethical requirements between different institutions ( Driscoll *et al.*, 2008). Inconsistency may arise as different IRBs may have different models in place or interpret guidelines differently ( Salt, 2019), which may be related to an absence of standardised forms, varying backgrounds and experiences of IRB members, and varying interpretations due to different culture and thinking at a cultural or regional level ( Gold & Dewa, 2005). Inconsistency may also relate to different views on methodological approaches ( Salt, 2019). Of particular relevance to intellectual disability research, Salt (2019) noted that inconsistency between IRBs may arise with regard to research with vulnerable populations, with different interpretations of vulnerability, ability to consent, and levels of risk.

However, as noted above, such challenges in the practical implementation of multi-site studies may remain even where policy and guidance provide for a single, centralised system, meaning that this type of research may still be “complex, bureaucratic and time consuming” ( Samir *et al.*, 2022: 16).

### Strategies for navigating the ethics process for multi-site studies

Several strategies have been identified in the literature to help researchers to overcome or mitigate the challenges that are presented in guiding multi-site studies through the ethical review process, especially where multiple IRBs are involved. Early advanced planning is important in anticipating and meeting issues that may arise during the application process ( Nichols *et al.*, 2019; Salt, 2019; Samir *et al.*, 2022). Proactive engagement with IRBs, representatives and key personnel may also help to identify and mitigate issues and concerns, as well as build better understandings of the proposed project in advance of formal applications ( Marquis-Gravel *et al.*, 2021; Salt, 2019; Samir *et al.*, 2022). Related to both planning and proactive engagement, good clear communication and the development of a communication plan were identified as important to facilitating a smoother application process ( Marquis-Gravel *et al.*, 2021; Nichols *et al.*, 2019). Also stemming from these strategies is the recommendation to develop good working relationships with local actors ( Marquis-Gravel *et al.*, 2021; Nichols *et al.*, 2019), providing education and training for all parties to better inform and prepare everyone about the study and related regulatory expectations ( Gold & Dewa, 2005; Nichols *et al.*, 2019), and to advocate for the study and its population ( Salt, 2019).

Above all, the development of a single, centralised and coordinated IRB system underpinned by an agreed framework at national or regional level is recommended as a strategy to overcome many of the challenges inherent in multiple IRB systems ( Cobb *et al.*, 2019; Driscoll *et al.*, 2008; Gold & Dewa, 2005; Samir *et al.*, 2022). This may involve a common application form and documentation ( Driscoll *et al.*, 2008; Gold & Dewa, 2005) and a shared electronic platform for easier management of information ( Cobb *et al.*, 2019; Gold & Dewa, 2005; Samir *et al.*, 2022). Such strategies may create a streamlined and integrated review process nationally and a mechanism to coordinate requests for more information ( Samir *et al.*, 2022) with a process of full ethical review by a primary IRB and subsequent expedited review by other IRBs involved ( Driscoll *et al.*, 2008).

As an example of what may be involved in moving towards a centralised review system, Cobb *et al.* (2019) reported on a National Institutes of Health (NIH) initiative in the US to establish a national IRB ‘reliance network’ to support national adoption of single IRB (sIRB) review. A reliance agreement is “a formal, written document that provides a mechanism for an institution engaged in research to delegate [IRB] review to an independent IRB or an IRB of another institution” ( John Hopkins University, 2023). Significant incentives to systematise and simplify reliance arrangements had been provided by a growth in regional and disease- or population-specific IRB reliance agreements, as well as movement towards increased use of sIRB review of multi-site research through changes in federal regulations ( Cobb *et al.*, 2019). The NIH funded development of a master IRB reliance agreement to support sIRB review across the Clinical and Translational Science Awards (CTSA) consortium ( Winkler *et al.*, 2015), which would be applicable across a wide range of human subject research, and later funded the Streamlined, Multisite, Accelerated Resources for Trials (SMART) IRB Platform ( NIH NCATS, 2022) to facilitate national adoption and implementation – supporting compliance with the 2018 NIH Single IRB Policy ( National Institutes of Health, 2016) and other sIRB arrangements. Following its introduction, the SMART IRB was widely adopted, leading to many institutions leaving previous arrangements in favour of the new platform; however, transition, adaptation, and learning remained an ongoing process as institutions navigated a shift in research oversight ( Cobb *et al.*, 2019).

### Aim of the paper

Within this context, this paper provides a case report and commentary of the process and challenges encountered by the Intellectual Disability Supplement to the Irish Longitudinal Study on Ageing (IDS-TILDA) through 15 years of engagement with IRBs in multiple sites in Ireland within the further context of the transition to the General Data Protection Regulation (GDPR) ( European Union, 2016) and the Health Research Regulations ( Government of Ireland, 2021) to support GDPR implementation in research.

## IDS-TILDA case report: Experience from 15 years of a multisite longitudinal study

The IDS-TILDA study originated in 2008 with three primary aims:

- To identify the principal influences on successful ageing in persons with an intellectual disability.
- To determine if these influences are the same or different to people ageing in the general population.
- To establish a baseline picture of a representative cohort of people aging with intellectual disability that may be followed longitudinally. ( McCarron *et al.*, 2011)

IDS-TILDA completed its fifth wave of data collection in 2023, and has reported every three years since the first Wave 1 report in 2011 ( Burke *et al.*, 2014; McCarron *et al.*, 2011; McCarron *et al.*, 2017; McCarron *et al.*, 2020). This review is of the ethics review process for IDS-TILDA through its first four waves of data collection, and includes an in-depth analysis of the ethical review process for Wave 4.

### IDS-TILDA ethical review process

The ethical approval process for IDS-TILDA has involved two main phases:

**Phase One: Preparation**

- Development and review of the initial study protocol.
- Development and review of study documentation including recruitment materials.
- Engagement with internal and external stakeholders including the IDS-TILDA Scientific Advisory Board and Steering Committee.
- Public and Patient Involvement (PPI) engagement of people with intellectual disabilities in the development and piloting of new study questions, and in the development and testing of accessible participant recruitment materials such as Participant Information Leaflets (PIL) and consent forms.
- Creation of a comprehensive packet of all related materials and procedures addressing study purpose, conduct, consent and confidentiality to support applications for ethical review.

**Phase Two: Application**

- Application for review and approval by the host academic institution, Trinity College Dublin (TCD) Faculty of Health Sciences (FHS) Research Ethics Committee (REC).
- Application for review and approval by individual disability organisations who support individuals and families who would be approached to participate in IDS-TILDA. In some cases, these applications were made to a delegated regional office of the Health Service Executive (HSE) rather than to the individual service provider.
- (Since Wave 4) Application to the national Health Research Consent Declaration Committee (HRCDC) for a Consent Declaration, facilitating the inclusion of participants with an intellectual disability who lack the capacity to provide personal explicit consent.

The focus in this paper is to describe the range of activities and effort involved in the second phase – the process of application for ethical approval through the host institution and the supporting disability organisations. A summary of the process for Waves 1–3 is provided first, followed by an in-depth analysis of the application process at Wave 4.

### The ethical review process for Waves 1–3

As a national study aiming to recruit a sample representative of the population of adults with intellectual disabilities aged 40+ years, IDS-TILDA was able to drawn on the National Intellectual Disability Database (NIDD) in Ireland as a sampling frame to randomly and, from the study’s perspective, blindly select the required number of participants at Wave 1 ( McCarron *et al.*, 2011). Overall, the application process for approval by the host academic institution at Wave 1 took two months from the formal application to granting of approval. The preparation of the application and supporting documentation took approximately six months prior to this, meaning an application period of up to eight months in total for approval within the academic institution.

Given that the NIDD is a register of people with intellectual disabilities who are supported and reported to NIDD by disability services in Ireland, recruitment of participants required engagement with those organisations, and this would include obtaining their ethical approval for the study. At Wave 1, a total of 138 individual service locations were involved in the study. As some IRBs managed ethical approval for multiple service locations, the study team was required to obtain separate ethical approval from 45 individual and regional IRBs. For example, approval by the IRB of one national service provider covered approval across 18 of its service locations. The subsequent approval process took a total of 18 months to complete ( McCarron *et al.*, 2011).

Having received ethical approval for Wave 1, for each subsequent Wave there was a requirement to complete a new ethics application for full review and approval by the host institution ethics committee (TCD REC). Following this approval re-affirmation was sought of the approval from the individual disability organisation IRBs who originally approved the study at Wave 1. For Wave 2 and 3, there was a willingness by individual organisation IRBs to consider the new written approval granted by the TCD REC for the next Wave, along with a letter from the Principal Investigator (PI) summarising changes between the Waves and requesting confirmation of continued support for the study. As such, a new full application for ethical approval from each individual IRB was not always required, greatly expediting the process at Waves 2 and 3. However, in some cases additional materials were requested and re-affirmation of approval was delayed particularly when requests had the potential to be inconsistent with the host institution approval terms for that Wave. At Wave 3, the overall process of obtaining reaffirmation lasted for six months. In some cases, the time needed to meet these local conditions delayed completion of data collection in those providers even though ultimately they were no major changes to what was approved by the TCD REC.

### The ethical review process for Wave 4

This section provides a detailed description of the process for ethical approval at Wave 4 of IDS-TILDA

***Initial Step: Application for ethical approval in the supporting academic institution.*** A formal application for ethical approval of Wave 4 was made to the TCD REC. Consistent with what was submitted in prior waves, the application included the completed REC application form along with 37 supporting documents, comprising different versions of letters to prospective participants, Participant Information Leaflets, consent forms, data collection tools, and other documents. There was significant prior work in reviewing existing and potential new measures and scales to include in the protocol, engagement with internal and external stakeholders including the Scientific Advisory Board and Steering Committee, and with individuals with intellectual disabilities to develop and review the protocol and accessible materials. Feedback from the TCD REC on the initial application required only minor amendments. A revised application addressing these points was submitted to the REC and full ethical approval was granted. The process was completed in two months.

A significant change at Wave 4 was the need to consider new regulations addressing the newly enforced GDPR regulations ( European Union, 2016) and the related Health Research Regulations ( Government of Ireland, 2021) guiding implementation of GDPR for health research:

- GDPR broadened the scope of data protection across the EU including definitions and categories of personal data, and the principles of privacy by default and design; and established new and enhanced rights for individuals including the right to be informed, rights of access, rectification, erasure, and objection, as well as stricter rules around consent ( Health Research Board, 2021).
- Data Protection Impact Assessment (DPIA) – A DPIA is a requirement “where processing operations are likely to result in a high risk to the rights and freedoms of natural persons … to evaluate, in particular, the origin, nature, particularity and severity of that risk” ( European Union, 2016).
- Data Processing Agreement (DPA) – A DPA must be put in place as GDPR “requires data controllers to sign a data processing agreement with any parties that act as data processors on their behalf” ( GDPR.EU, 2023).
- Stricter rules around consent included the requirement to obtain explicit consent directly from individuals to participate in health research.
- Consent Declaration – In the absence of explicit consent from individuals, such as where they lack the capacity to provide informed consent, a case may be made to include individuals on the basis of public interest. In such cases, an application for a Consent Declaration is required – through the Health Research Consent Declaration Committee (HRCDC) in Ireland ( HRCDC, 2023).

While not a requirement when the application for ethical approval was originally submitted to the TCD REC, a DPIA and a number of DPAs were subsequently developed and approved by the TCD Data Protection Officer (DPO) ahead of the start of W4 recruitment and data collection. Similarly, a Consent Declaration was obtained from the HRCDC to facilitate data collection with participants who did not have the capacity to give explicit consent.

***Application for ethical approval at supporting disability services.*** Following confirmation of approval by the TCD REC in January 2019, preparations for ethical review by the supporting disability services across the country began in February 2019 with administrative support and communications to advance the process. The number of individual IRBs approached had increased from 45 in Wave 1 to 48 in Wave 4 with the addition of new services participating in the study. As with Wave 1, some of these IRBs managed ethical approval for multiple service locations, with the total number of study sites at 101 for Wave 4.

**Process and timeframe for ethical approval by supporting disability services:**

Beginning at the end of January 2019, initial contacts were made by phone and email to establish and re-establish contacts with key personnel at each site, and to alert them of the pending application for ethical approval. Formal requests for the affirmation of ethical approval were issued to all 48 supporting organisations by May 2019. Ethical approval from these individual organisations was sought on the basis of the approval already granted by the TCD REC, as well as the organisations’ established knowledge of and relationship with the study, which had been built through previous ethical approval and engagement in Waves 1–3 of the longitudinal study. A copy of the TCD approval was sent to each organisation along with a letter from the PI outlining changes to the protocol since the previous Wave, and seeking renewed affirmation from the organisation for the current Wave. A letter from the TCD Data Protection Officer stating that the study adhered to GDPR and HRR requirements was also provided.

Table 1 summarises the ethical application requirements utilised by participating organisations in Wave 4 and the length of time needed to obtain approval. Of the 48 individual organisations supporting the study, 43 (90%) granted ethical approval on the basis of the received TCD REC approval and the clarification of changes summarised in the letter from the PI. Two organisations required a full application for ethical approval be submitted using their own application/proposal form. One organisation required a full application (as if this was a new study) using a Standard Application Form (SAF) alone – the SAF is a standard ethics form developed by the Health Service Executive (HSE) in Ireland and used by a number of organisations nationally. One organisation required a completed SAF and a DPIA completed. And one organisation required a completed SAF with a DPIA and (following review and requests for changes) specific participant documents (e.g., PILs and consent forms) and other documents tailored to the requirements of the organisation.

**Table 1.** Ethical application requirements for participating organisations (Wave 4).

Type of Application	Number of Orgs	Informal Start Date	Formal Start Date (Official Request)	End Date	No. of Contacts Average (Sum)	Weeks to Approve (formal): Average (Range)
**Affirmation letter & TCD approval**	43	Jan-2019	07.05.19	09.03.20	5 (235)	7 (<1 to 44)
**Organisational form**	2	Jan-2019	08.05.19	14.06.19	6 (12)	5 (5)
**Standard Application Form (SAF)**	1	Jan-2019	09.05.19	14.05.19	4 (4)	1
**SAF & DPIA**	1	Jan-2019	17.05.19	24.05.19	5 (5)	1
**SAF & DPIA & Tailored Documents**	1	Jan-2019	17.05.19	08.04.20	70 (70)	47
**Total**	**48**	**Jan-2019**	**07.05.19**	**08.04.20**	**7** **(326)**	**8** **(<1 to 47)**

For the large majority of organisations, therefore, approval was obtained on the basis of prior TCD approval and a supporting letter requesting affirmation. Among these 43 organisations, the majority (60%, 26/43) gave approval within four weeks, while a quarter (23%) took longer than eight weeks to approve. The average period to approve among organisations who accepted the affirmation letter was just 7 weeks. However, this was enabled by the significant amount of time not included in this figure that was spent by the Project Manager liaising with services and IRBs prior to the formal request for approval. Additionally, there was a wide range within this 7-week average – from same-day approval by some organisations, up to 44 weeks for others. The five organisations who took longest to approve using the affirmation letter took between 21 and 44 weeks to grant approval.

For the organisations which required more than the affirmation letter:

- The two organisations who required an application for ethical approval using their own application/proposal form each took five weeks to grant approval.
- The organisation that required a full application using the SAF granted approval within less than one week.
- The organisation that required a completed SAF and a DPIA completed also granted approval within one week.
- The organisation that required a completed SAF with DPIA and specific study documents tailored to its requirements took 47 weeks to approve from the formal application date.

**Level of engagement and follow-up with supporting disability services:**

Table 1 also outlines the number of contacts with organisations throughout the ethics review process. Contact included emails and phone calls throughout the process. This shows that there was an average of seven contacts made with each organisation to achieve ethical approval, with a total of 326 contacts for the 48 organisations.

**Outcomes/impact of ethical review by multiple IRBs**

Almost 9 in 10 organisations/IRBs granted approval within 13 weeks, facilitating the commencement of data collection with individuals supported by their services within the planned study timeframe (September 2019). For six organisations/IRBs who took longer to formally approve (21–47 weeks), the commencement of data collection with individuals they supported was delayed between one and eight months. Despite the time taken to grant ethical approval, 47 of 48 organisations/IRBs (98%) were satisfied to proceed without any changes to the study documentation, including four of the five IRBs who required that new applications be made at Wave 4. There were no changes requested to the study protocol. In one case there was a need to change the study PILs and consent forms to meet their specific requirements but not in ways that had any impact on the study and its processes beyond the delayed start of data collection. Therefore, a critical impact of the process was the delay to the commencement of data collection within a small number of organisations. In the case of one organisation, the extent of the delay in their approval process resulted in the involvement in only part of the study by existing research subjects, as well as no recruitment of new participants from the organisation during Wave 4.

## Discussion

The experience of IDS-TILDA described in this case study reflects, in many ways, prior experiences and challenges presented in the literature of multi-site studies seeking ethical approval from multiple IRBs. Supporting previous studies, we found that the ethical review process with multiple IRBs increased the degree of complexity of the process, for example requiring different formats of application, as well as the level of bureaucracy associated with this ( Driscoll *et al.*, 2008; Marquis-Gravel *et al.*, 2021; Samir *et al.*, 2022; Smith *et al.*, 2019). Related to this, we found that engagement across multiple IRBs also greatly increased the complexity and amount of communication required ( Smith *et al.*, 2019), considering that 48 IRBs were involved rather than a single central entity. Similarly, findings here of the additional time and resource implications of engaging in a process with multiple IRBs support previous findings ( Driscoll *et al.*, 2008; Gold & Dewa, 2005; Greene & Geiger, 2006; Marquis-Gravel *et al.*, 2021; Racine & Bracken-Roche, 2019; Salt, 2019; Samir *et al.*, 2022; Smith *et al.*, 2019). For IDS-TILDA, this resulted in delays of up to 11 months for a small number of organisations – which had a knock-on effect for the study as a whole for completing data collection and beginning data cleaning and analysis. Resource implications were largely related to the significant increase in contacts required to progress the process, and the efforts by study personnel entailed in developing applications and supporting documentation in different formats for different sites. Given how these factors ultimately delayed and restricted the study, our findings concur that, in some ways, this may present a barrier to the conduct of ethical research rather than supporting it ( Gold & Dewa, 2005; Menikoff, 2010; Samir *et al.*, 2022; Silberman & Kahn, 2011; Smith *et al.*, 2019).

Interestingly, one of the key challenges of multiple IRB processes that was highlighted in the literature – the risk of variability and inconsistency across different IRBs ( Driscoll *et al.*, 2008; Gold & Dewa, 2005; Marquis-Gravel *et al.*, 2021; Salt, 2019) – did not present as much of a challenge for IDS-TILDA. While there was a degree of variability in the application process for a small number of IRBs, in terms of requiring use of their own forms or additional requirements, this did not carry through to varying or inconsistent interpretations of the study by different IRBs – with no changes requested to the protocol or proposed study methods as a result of the ethics process. However, this may be related to the work already done in previous waves and to the established and ongoing communication with key personnel, something which non-longitudinal studies may not benefit from and therefore may be unable to avoid this type of variability in the outcomes of their ethics processes.

Notwithstanding the significant challenges encountered, the example of IDS-TILDA also supports some previously identified strategies for overcoming these challenges. It confirmed how strategies including early advanced planning ( Nichols *et al.*, 2019; Salt, 2019; Samir *et al.*, 2022), proactive engagement with IRBs ( Marquis-Gravel *et al.*, 2021; Salt, 2019; Samir *et al.*, 2022), good communication including a clear plan ( Marquis-Gravel *et al.*, 2021; Nichols *et al.*, 2019), and investing time in developing relationships with local agents ( Marquis-Gravel *et al.*, 2021; Nichols *et al.*, 2019) were not only beneficial but essential to achieving ethical approval across multiple IRBs.

Therefore, many of the previously identified challenges and strategies are echoed in the experience of IDS-TILDA. However, the case of IDS-TILDA presents a unique example in some important ways, due to being a multi-wave longitudinal study which has engaged many of the same participants and supporting organisations and IRBs over several years. This has provided an insight to the potential for a single IRB system, given that IDS-TILDA has been able to utilise some of the critical aspects of a single IRB, albeit without a formal system being in place. For example, in Wave 4 of the longitudinal study, IDS-TILDA did not need to complete a new ethics application for the majority of IRBs involved in the study – who granted approval on the basis of prior ethical approval from the host academic institution along with an update on protocol changes from the study PI. However, as reported, this simplified process did not in itself guarantee an expedited overall process. While the process was expedited for many, for others this was not the case, and a significant amount of follow-up was required before approval was granted. Another element that IDS-TILDA was able to utilise was the Standard Application Form (SAF) that is used by several disability services in their ethics application process. Three of the five IRBs who required a new ethics application at Wave 4 used the SAF and this showed the additional potential for simplification and avoidance of duplication, should such a form be used in the context of a single IRB system.

A critical point here is that, while experiencing some frustrations, IDS-TILDA was nonetheless able to benefit from some of the features of a single IRB system. However, without a formal system in place, it was primarily able to achieve these benefits because of the significant amount prior work completed in earlier waves of the longitudinal study, particularly at the start, which laid the groundwork for a more expedited process in later waves of the study – particularly in the extensive amount of time spent by the PI and Project Manager in developing and maintaining the relationships with key personnel which would be critical to supporting the ethics approval process later on. For other studies, which are once-off in nature rather than longitudinal, this benefit is unlikely to be accrued. For many, this may result in a curtailing of important research given that potential delays and other issues may impact the feasibility of such studies ( Gold & Dewa, 2005; Menikoff, 2010; Samir *et al.*, 2022; Silberman & Kahn, 2011; Smith *et al.*, 2019).

This experience highlights the potential for a single IRB system, the benefits of which may be available both to once-off studies and multi-wave longitudinal studies. The added value that a more formalised single IRB system may be assumed to bring is that it may both simplify *and* expedite the ethics approval process across multiple study sites. However, some examples in the literature highlight that much work remains to optimise the operation of such systems even after the regulatory framework has been put in place ( Driscoll *et al.*, 2008; Marquis-Gravel *et al.*, 2021; Nichols *et al.*, 2019; Samir *et al.*, 2022) and the system has been established ( Cobb *et al.*, 2019).

Nonetheless, the case study of IDS-TILDA highlights the potential for moving towards a single IRB system through more widespread utilisation of the SAF. However, for multi-site studies to fully realise the benefits of a single IRB system, agreement is also needed on a centralised process whereby initial ethical review by the supporting academic institution or other designated body is subsequently adopted by individual organisations within the system. To achieve this, coordination and regulation is needed by the authority funding the organisations who are the gatekeepers for research that may be conducted.

The implementation of GDPR and the Health Research Regulations in Ireland in recent years has introduced a higher degree of standardisation in research ethics, and this may provide an opportunity to standardise and centralise the ethical approval process for the benefit of all – for researchers, for support organisations, and ultimately for people with an intellectual disability and other populations under investigation.

## Data Availability

Data cited in this paper were derived from IDS-TILDA administrative data. To protect the confidential information of persons and organisations involved, this data cannot be made available publicly.
