# Supplementary material for: IRB challenges in multisite studies: A case report and commentary from the Intellectual Disability Supplement to the Irish Longitudinal Study on Ageing (IDS-TILDA)
Source: HRB Open Res. 2024 Feb 12;7:3. [Version 1] doi: 10.12688/hrbopenres.13854.1 (PMC11109535; doi:10.12688/hrbopenres.13854.1)
Supplement: Peer review history

# Reviewer report: Reviewer response for version 1

**DOI:** 10.21956/hrbopenres.15182.r39092

**Published:** 2024-05-09

**Authors:** Roy Elbers

**Affiliations:** 1 Department of General Practice, Intellectual Disability Medicine, Erasmus MC, University Medical Center, Rotterdam, The Netherlands

**Elaboration on questions answered ‘no’ or ‘partially’**

*Are sufficient details of methods and analysis provided to allow replication by others?*

 The authors do not provide a detailed description of their methods, but readers can derive them from the result presented in the paper. Given the purpose of the study, replication of their results is not likely to be an aim for future studies.

*Are all the source data underlying the results available to ensure full reproducibility?*

 Given the confidential nature of the data (and difficulty of de-identification), the authors choose not to provide access to their data. This makes sense to me.

**Other comments to the editor and authors**

 The authors wrote a well-written manuscript that describes their experiences in obtaining ethical approval from different institutional review boards for the longitudinal IDS-TILDA study.

 I have only a few minor comments that may improve the presentation of the results.

*Page 7, Table 1 abbreviations*

 Although the authors spelled out all abbreviations in the manuscript, readability of the table may improve if they spell out the abbreviations (TCD, SAF and DPIA) in a footnote to the table.

*Page 7, ‘Additionally, there was a wide range within this 7-week average... ...44 weeks for others’*

 Given this wide range, and extreme outliers in weeks to approval, I am not sure if the average is an informative central tendency. The median numbers of weeks may be a more representative summary of these data.

Is the work clearly and accurately presented and does it cite the current literature?

Yes

If applicable, is the statistical analysis and its interpretation appropriate?

Not applicable

Are all the source data underlying the results available to ensure full reproducibility?

No

Is the study design appropriate and is the work technically sound?

Yes

Are the conclusions drawn adequately supported by the results?

Yes

Are sufficient details of methods and analysis provided to allow replication by others?

Partly

Reviewer Expertise:

Research methods, Meta-analysis, Intervention studies, Intellectual Disability, Rehabilitation

I confirm that I have read this submission and believe that I have an appropriate level of expertise to confirm that it is of an acceptable scientific standard.

# Reviewer report: Reviewer response for version 1

**DOI:** 10.21956/hrbopenres.15182.r39095

**Published:** 2024-05-09

**Authors:** Caroline Egan

**Affiliations:** 1 University College Cork, Cork, County Cork, Ireland

Thank you for inviting me to review this paper which provides a case report and commentary on the process and challenges which the IDS-TILDA encountered navigating institutional and disability service research boards.

 The paper is well written with a clear focus. The introduction is comprehensive providing a strong rationale for this case report. An international and national focus and context is provided.

 The case report details the resources and overall complexity which the multisite engagement with research committees entailed, describing the individual stages of the application process within the 4 waves of IDS-TILDA.

 The paper encourages dialogue on this issue, which is delaying or indeed impeding the research process. The findings presented here do indicate that the current approach presents a barrier to the conduct of ethical research rather than supporting it.

 A single application to the institutional research board, and centralised process in this scenario does appear reasonable. However stringent safeguards under a regulatory framework is required.

 Further international research evaluating resources and approaches (Serdoz et al. 2022 (Ref 2) ; Cobb et al. 2019) [Ref 1] to a centralised review system is required.

 To conclude this paper makes a strong argument highlighting the potential for moving towards a single IRB system. 

 Cobb N, Witte E, Cervone M, *et al.*: The SMART IRB platform: A national resource for IRB review for multisite studies. *J Clin Transl Sci.* 2019; **3**(4): 129–139[Ref 1]

Is the work clearly and accurately presented and does it cite the current literature?

Yes

If applicable, is the statistical analysis and its interpretation appropriate?

I cannot comment. A qualified statistician is required.

Are all the source data underlying the results available to ensure full reproducibility?

Yes

Is the study design appropriate and is the work technically sound?

Yes

Are the conclusions drawn adequately supported by the results?

Yes

Are sufficient details of methods and analysis provided to allow replication by others?

Yes

Reviewer Expertise:

Nursing, Intellectual disability, Ageing

I confirm that I have read this submission and believe that I have an appropriate level of expertise to confirm that it is of an acceptable scientific standard.

## References

[1] : The SMART IRB platform: A national resource for IRB review for multisite studies. *J Clin Transl Sci* .2019;3(4) : 10.1017/cts.2019.394 129-139 10.1017/cts.2019.394 31660237 PMC6798516

[2] : The IRB Reliance Exchange (IREx): A national web-based platform for operationalizing single IRB review. *J Clin Transl Sci* .2022;6(1) : 10.1017/cts.2022.376 e39 10.1017/cts.2022.376 35574155 PMC9066316

# Reviewer report: Reviewer response for version 1

**DOI:** 10.21956/hrbopenres.15182.r38269

**Published:** 2024-03-14

**Authors:** Nichelle Cobb

**Affiliations:** 1 Association for the Accreditation of Human Research Protection Programs, Washington, District of Columbia, USA

This article by McCausland et al adds an important case study to the literature on how the use of multiple ethics committees, or institutional review boards (IRBs), affects time to study start up and associated costs. Additionally, this paper includes a thoughtful exploration of how the single IRB model could be used to streamline the ethics review process within the Irish ethical review system and the factors that would contribute to a more efficient process. The paper’s Introduction section provides a robust overview of the motivations for moving to a single IRB model and challenges with multiple ethics review processes for multi-site research. Finally, the Discussion outlines potential helpful directions to pursue to address these challenges rooted in the investigators’ unique experience with a long-term research project.

 What follows are questions or observations I had in reaction to the article. Admittedly, some of the questions arose due to this reviewer’s lack of familiarity with Irish human subjects research regulation and processes. I recognize the article is not intended for a primarily American audience but think this paper will be of interest to US readers. Further, the article draws upon the American single IRB model as part of the discussion, which is why I have included suggestions some specific comparisons and contrasts with the US system that could further flesh out an already robust paper.

- The authors noted in the description of the ethical review process for Waves 1-3 that “the preparation of the application and supporting documentation took approximately six months prior to this, meaning an application period of up to eight months in total for approval within the academic institution.” **It would be helpful to the reader to understand what the preparation for review entailed and why the application process took approximately six months so that other potential areas for streamlining the review process might be identified. **
- ****How is the review of compliance with GDPR in the Irish system approached? Is it centralized? How has standardization of interpretation been achieved?** The paper suggests that “the implementation of GDPR and the Health Research Regulations in Ireland in recent years has introduced a higher degree of standardisation in research ethics, and this may provide an opportunity to standardise and centralise the ethical approval process for the benefit of all.” **For the sake of comparison, in the US, a near equivalent review for the GDPR is assessment for compliance with the Health Information Portability and Accountability Act (HIPAA) Privacy Rule. US IRBs can be empowered to take on HIPAA Privacy Rule review functions, such as considering and granting waivers of the requirement to obtain authorization for the use of protected health information (PHI) in a research study. Thus, HIPAA Privacy Rule reviews can be combined with IRB review. There has been a reluctance for some IRBs to perform HIPAA Privacy Rule reviews for other organizations because each institution’s interpretation of HIPAA related to their patients’ PHI varies. The lack of a centralized HIPAA Privacy Rule review and variation in interpretation of the HIPAA Privacy Rule continues to impede the efficiency of single IRB review in the US because of the lack of a centralized process.
- The transition to single IRB model in the US has clarified the distinction between IRB responsibilities and those of an institution’s human research protection program (HRPP). In many cases, IRBs served as gatekeepers for a range of ancillary requirements their institutional HRPPs imposed that had to be met before a study could be activated. For example, IRBs often held their approvals until other ancillary reviews were completed, such as conflict of interest assessments, contract review, radiation or biological safety reviews, and data security reviews. With a transition to the use of an external single IRB for research, institutions have had to develop new HRPP processes for ensuring that these ancillary reviews are completed before a study can be activated (Ref [1]). Additionally, it has become clear that although single IRB review has the potential to streamline a significant component of the process required to open studies (i.e., ethics review), IRB review was not the only reason and often not the significant reason for such delays (e.g.,Ref [2]). **This leads to the question for the IDS-TILDA** **of whether, aside from IRB review, the research was subject to other ancillary reviews at institutions, and, if so, if those reviews were intertwined with the IRB review process at the institution (i.e., the institutional IRBs served as gatekeepers for other reviews) such that the timeframes provided solely reflect IRB review at each institution?** Based on this commenter’s experience, IRB review is often conflated with other ancillary HRPP reviews, so parsing IRB and other required reviews is important to identifying the bottlenecks that delay study activation and understanding the potential benefits and limitations of single IRB review.
- One component of single IRB review in the US that can significantly affect the efficiency of the review process is the expectation for single IRBs to obtain local context information from each institution that relies on their reviews because no standardized process for the collection of local context information or agreement regarding what is considered local context exists. Recently, OHRP issued guidance about the use of single IRB, which defines “local context” as “generally referring to local circumstances, preferences, and variability, and could include such factors as culture and language, geography, socioeconomic factors, the professionals conducting the research, the institutions where the research will be conducted, or local standards of care.” ( https://www.hhs.gov/ohrp/regulations-and-policy/requests-for-comments/draft-guidance-use-single-institutional-review-board-for-cooperative-research/index.html). An aspect of local context that can be particularly critical for US IRBs to capture in order to adequately protect the rights and welfare of research participants is variation in state laws that can affect research, especially influence risks to participants. The need to collect and apply local context information as part of the ethics review, which sometimes encompasses the outcomes of ancillary reviews, has affected the efficiency of the single IRB model (Ref [1]). **Perhaps the article’s authors could address how the concept of local context plays out for a multi-site study in Ireland, including whether it is relevant to that system and, if so, how it might parallel or differ from the US approach. Further, if local context is relevant, how might the collection and review of that information affect the review process and time to study activation? **
- Another review process the research underwent that the article notes, which appears to be centralized,  was for a Consent Declaration from the HRCDC. In the US, IRBs can grant waivers of informed consent or allow legally authorized representatives to provide consent on behalf of individuals unable to provide informed consent form themselves (often referred to as surrogate consent), which appear to be analogous to the Consent Declarations that the HRCDC can issue. In a multi-IRB review situation, IRBs can disagree with each other about when a waiver of informed consent can be granted or how surrogate consent is implemented, which can be frustrating for researchers and impose inconsistent conditions that can affect research participant enrollment. A benefit of a single IRB is the elimination of inconsistent decisions, which this article notes. One other potential area for exploration, however, is whether a centralized process for requesting Consent Declarations separate from the ethics review process presents challenges to efficiency or the potential for a disagreement to occur between the HRCDC and IRBs reviewing the research. **It would be helpful to readers for the authors to describe how the HRCDC and IRB functions differ and whether there is any overlap, and thus potential for a conflict in determinations.**
- In the American system, whether an organization is considered *engaged* in non-exempt human subjects research is a concept key to determining whether IRB review is required for a study conducted or supported by the US Department of Health and Human Services (HHS), which funds a significant amount of research. Organizations that are engaged in non-exempt human subjects research remain responsible for the protection of the rights and welfare research participants and ensuring compliance with an IRB’s determinations, whether internal or external to their organization. Thus, it is incumbent on an organization to have processes to meet their responsibilities as relying organizations (Cobb, N and Jones, M. 2020. Responsibilities of the Relying Institution. In: Hurley et al (Eds.) Institutional Review Board Management and Function, 3 ^rd^ edition). **What might the authors think about how distribution of responsibilities between the IRB and relying institutions might pose challenges should Ireland adopt a single IRB system. This question might be beyond the scope of the current paper, but it may be an issue the authors could include as a consideration in their Discussion section.** When the researchers are not employees or agents of an institution at which research is conducted, the location of the research is considered a performance site and if that site has a local IRB, it would not be obligated by regulation to review the research. Instead, performance sites often conduct a limited review to determine whether they support the conduct of the study at their site and whether any institutional policies apply that the researchers must follow. Institutions that are not engaged in human subjects research are not required to obtain IRB review. The US Office for Human Research Protections (OHRP) issued guidance ( https://www.hhs.gov/ohrp/regulations-and-policy/guidance/guidance-on-engagement-of-institutions/index.html) that identifies activities that engage or do not engage an institution in non-exempt human subjects research. For example, conducting activities that involve patients at an institution does not automatically engage that institution in human subjects research, particularly if the researchers are not agents or employees of that institution. **Would the individual disability organizations that support individuals and families who were approached to participate in IDS-TILDA be considered participating sites rather than engaged in human subjects research in the US system? It would be interesting and helpful to know what triggers the need for IRB review within the Irish ethics review process and how that influences the number of IRBs required to review a study and what they review. **
- One last comparison with the American system that might be of interest to consider when thinking about streamlining review processes under the single IRB model is the potential time savings for continuing reviews and amendments to the research protocol. In the US, the effort related to the single IRB model is frontloaded with execution of a reliance agreement and the initial review of the study. US regulations require documentation when an organization relies on the IRB of another organization to provide review of non-exempt research it engages in. As the article’s authors note, considerable progress has been made in the US to reduce the time to execute a reliance arrangement, especially with the use of the SMART IRB Agreement. As noted above, the collection of local context information and completion of ancillary reviews at relying institutions can slow down the single IRB review process at the time a study undergoes initial review. However, the IRB review process can be less cumbersome and faster for the consideration of protocol amendments (changes in research) and continuing review for multi-site studies using the single IRB model. Continuing review is an at least annual review required for most research that presents more than minimal risks. **This article includes valuable insights into the review process and timelines when transition between waves for IDS-TILDA, which made this reviewer wonder what the process is in the Irish system for review of protocol amendments and whether there are any analogous requirements for continuing review. If so, would the authors view the single IRB model as providing benefits for these types of reviews as well?**

Thank you for the opportunity to engage with the authors of this stimulating article.

Is the work clearly and accurately presented and does it cite the current literature?

Yes

If applicable, is the statistical analysis and its interpretation appropriate?

Not applicable

Are all the source data underlying the results available to ensure full reproducibility?

Yes

Is the study design appropriate and is the work technically sound?

Yes

Are the conclusions drawn adequately supported by the results?

Yes

Are sufficient details of methods and analysis provided to allow replication by others?

Yes

Reviewer Expertise:

single IRB review, informed consent in research, human subjects regulations, IRB processes

I confirm that I have read this submission and believe that I have an appropriate level of expertise to confirm that it is of an acceptable scientific standard.

## References

[1] : Implementation of single IRB review for multisite human subjects research: Persistent challenges and possible solutions. *J Clin Transl Sci* .2023;7(1) : 10.1017/cts.2023.517 e99 10.1017/cts.2023.517 37250991 PMC10225260

[2] : Drivers of Start-Up Delays in Global Randomized Clinical Trials. *Ther Innov Regul Sci* .2021;55(1) : 10.1007/s43441-020-00207-2 212-227 10.1007/s43441-020-00207-2 32959207 PMC7505220
